# Winnow-KAN: single-cell RNA-seq location recovery with small-gene-set spatial transcriptomics

**DOI:** 10.1186/s12859-025-06243-9

**Published:** 2025-08-12

**Authors:** Yuyang Zhang, Qihuang Zhang

**Affiliations:** Department of Epidemiology, Biostatistics and Occupational Health, McGill University, Montreal, Quebec Canada

**Keywords:** Cell mapping, Deep learning, Kolmogorov-Arnold network, Single-cell RNA-seq, Spatial transcriptomics

## Abstract

Keywords: Cell mapping, Deep Learning, Kolmogorov-Arnold network, Single-cell RNA-seq, Spatial transcriptomics.

Single-cell RNA sequencing (scRNA-seq) has revolutionized our understanding of cellular heterogeneity. However, its collection process prevents the investigation of tissue organization due to the lack of spatial origins for the cells. Recent advances in computational methods have addressed this gap by leveraging spatial transcriptomics, which simultaneously profiles gene expression and spatial coordinates. While these state-of-the-art methods demonstrate excellent performance in cell location recovery, their effectiveness is often specific to the particular pair of scRNA-seq and spatial transcriptomics datasets used, limiting their scalability to larger datasets and generalizability to external query scRNA-seq data. In this study, we demonstrate the feasibility of leveraging a novel model architecture to address the redundancy in scRNA-seq datasets and facilitate prediction with a much smaller set of genes. We present Winnow-KAN, a method designed to reduce the number of required gene variables in cell-mapping tasks. Built on a modified structure of the Kolmogorov-Arnold Network, Winnow-KAN leverages the Kolmogorov-Arnold Representation Theorem to facilitate location predictions using fewer features than those required by multi-layer perceptron-based methods. Winnow-KAN includes a selector layer that reduces the size of the gene set used for prediction, enabling the model to recover query scRNA-seq data with performance comparable to MLP-based approaches that rely on the full gene set. We benchmarked Winnow-KAN using multiple datasets generated from brain and cancer tissues, derived from platforms such as Visium and MERFISH.

**Supplementary Information:**

The online version contains supplementary material available at 10.1186/s12859-025-06243-9.

## Background

The emergence of single-cell RNA sequencing (scRNA-seq) has significantly advanced our understanding of cellular heterogeneity by enabling the profiling of gene expression at a single-cell resolution. This breakthrough allows researchers to explore the molecular complexity of individual cells, revealing previously obscured cellular diversity that was often masked in bulk analyses [17]. However, the power of scRNA-seq is hampered by a major limitation: the dissociation of cells during data collection, which removes their spatial context. As a result, while scRNA-seq provides valuable information in a cellular level, it does not offer insights into the spatial organization of tissues which is essential for studying tissue architecture and the distribution of cell types, both of which are crucial for understanding disease progression.

To address this limitation, there has been growing interest of developing computational methods to recover spatial information for the scRNA-seq data. Dating back to the early work of Seurat [26], spatial marker genes have been used to map cells to specific regions indicated by these markers, enabling the reconstruction of spatial information. However, as some regions lack identifiable spatial marker genes, it might be impossible to map cells to those regions [21, 34]. Additionally, even when spatial marker genes exist, they may not be captured in single-cell data due to the limitations of sequencing capacity [9, 25].

Recent advances in spatial transcriptomics (ST) have provided an exciting solution as gene expression can be profiled while preserving their spatial origin in the tissues [27]. Such data enable the direct mapping of gene expression to tissue architecture, offering insights into how spatial organization is associated with gene expression within a native environment [31]. This naturally lends itself to the formulation of a supervised learning framework for location recovery, where spatial transcriptomics serves as the training set to learn the relationship between gene expression and spatial locations, which can then be applied to query scRNA-seq data. Tools like Tangram [3], NovoSpaRc [22], and spaOTsc [5] use optimal transport algorithms to map scRNA-seq to spatially transcriptomics, allowing for the integration of both datasets. However, these methods treat each mapping instance independently and require rerunning the process for every new query scRNA-seq dataset. This limits scalability when multiple scRNA-seq datasets need to be processed, even if they originate from the same tissue context. A more recent method, CeLEry [34], introduces a deep learning model that applies supervised learning to spatial transcriptomics data, providing a pretrained model that can be applied to multiple query datasets. CeLEry supports two types of spatial location recovery: domain recovery, which maps cells to broader tissue regions, and coordinate prediction, which identifies specific spatial coordinates. While CeLEry presents a significant advancement in its ability to process large-scale data and generalize across multiple datasets, its design typically relies on a large number of genes as input features. In practice, this can introduce challenges when applying the pretrained model to query datasets, as scRNA-seq data often vary in gene coverage due to differences in sequencing platforms or preprocessing pipelines, limiting compatibility between the training and query sets.

Due to the characteristics and limitations of the existing methods, they are often restricted by the high dimensionality of both single-cell and spatial transcriptomics datasets, as a large number of genes are profiled [31]. Firstly, the large number of genes in these datasets increases the risk of model overfitting, especially when applying pretrained models to new tissues or experimental conditions. Many genes do not exhibit spatially specific expression patterns and contribute primarily as noise, while others provide redundant information due to overlapping spatial patterns. Second, to ensure the generalizability of pre-trained models, the selected gene set should be minimal in size to guarantee its compatibility with the query data. However, this constraint can, in return, reduce the predictive power of the model, potentially leading to suboptimal performance. Therefore, filtering out non-informative genes while retaining those that provide the most relevant information for spatial location prediction is a critical step in improving model performance.

To address these challenges, we introduce Winnow-KAN, a novel deep-learning framework designed to streamline spatial transcriptomics analysis by employing a modified version of Kolmogorov-Arnold Networks (KAN, Liu et al. [19]). Unlike traditional deep neural networks (DNNs) based methods, such as CeLEry, which rely on multi-layer perceptrons (MLPs) and are often criticized for their lack of interpretability. KAN leverages the Kolmogorov-Arnold Representation Theorem [16] to approximate complex, non-linear functions with fewer features. Meanwhile, rather than learning a geometry-invariant representation, Winnow-KAN is designed to map the cells in scRNA-seq data onto a biologically matched spatial reference, treating the spatial transcriptomics sample as a localization template consistent with prior cell mapping formulations (e.g., Zhang et al. [34]). This unique architecture not only enhances computational efficiency but also provides interpretable insights by identifying key variables that contribute to prediction through learned variable importance during training [19]. Winnow-KAN incorporates a selector layer that simultaneously helps to predict spatial information and identifies the most influential genes throughout the prediction. By narrowing the focus to a smaller, more meaningful set of genes, Winnow-KAN enables both accurate spatial prediction and biologically interpretable insights into spatial organization. Unlike differential expression (DE) analysis, which identifies genes with significant mean expression differences between regions, Winnow-KAN selects genes based on their predictive value. As a result, it can uncover biologically important genes that may not rank highly in DE analyses but nonetheless capture critical spatial information relevant for localization tasks. These approaches are thus complementary, offering distinct yet informative views of gene relevance.

In this study, we demonstrate the effectiveness of Winnow-KAN through extensive benchmarking on multiple datasets, including brain and cancer tissues generated from platforms such as Visium and MERFISH. Our results show that Winnow-KAN can accurately recover spatial layers and predict 2D cell coordinates with high precision, even when using a significantly reduced set of genes. Furthermore, Winnow-KAN outperforms traditional MLPs and other state-of-the-art methods in both prediction accuracy and gene selection, offering a powerful tool for advancing spatial transcriptomics research.

## Methods

### Overview of Winnow-KAN

**Fig. 1:**
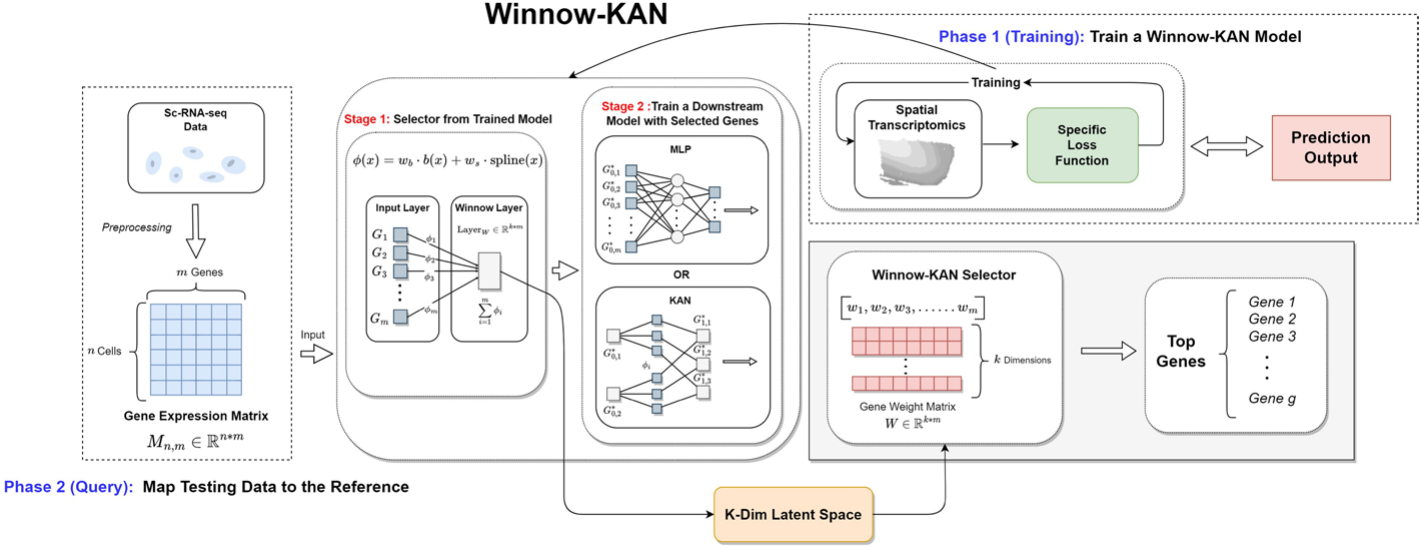
Workflow of Winnow-KAN: Winnow-KAN has two phases. The training phase begins with spatial transcriptomics data, where a supervised model is trained to predict spatial information (e.g., 2D coordinates or spatial domains) from gene expression using a task-specific loss function. The training process is organized into two sequential and independent blocks. In Stage 1, the Winnow Block is trained to processes the input expression data and projects it into a lower-dimensional latent space by feature selection via a learned weight matrix, identifying the genes most relevant to the spatial prediction task. Once the informative genes are selected, the input data are reduced to this subset. In Stage 2, a Prediction Block, which can be either a multi-layer perceptron or a Kolmogorov-Arnold Network (KAN) block depending on the implementation, is trained from scratch using only the selected gene set to learn the mapping from expression to spatial labels. Once trained, the model can be applied to scRNA-seq data (query data). The scRNA-seq expression matrix is passed through the same Winnow-KAN architecture to produce predicted spatial locations or domain labels. The trained selector layer also outputs a ranked list of the most informative genes, enabling interpretable insights into spatial patterning and supporting downstream biological discovery

The key concept of Winnow-KAN (Fig. 1) is to construct a deep learning model that learns the relationship between gene expression and spatial location using spatial transcriptomics data (reference data). Once trained, the model takes the gene expression profile of a cell from scRNA-seq data (query data) as input and predicts its spatial location. The predictive model incorporates a modified Kolmogorov-Arnold Network (KAN) originally proposed by Liu et al. [19], leveraging its ability to approximate nonlinear functions and provide model interpretability via variable selection. This framework allows our method not only to outperform multi-layer perceptron (MLP) in predicting spatial locations using fewer features, but also to identify genes that are most important for prediction, aiding the discovery of spatially informative genes. Our modifications address the original KAN’s computational inefficiencies and enable support for GPU acceleration via CUDA (as discussed in Section A.3 of Supplementary Materials).

### Spatial information prediction model

Let $X_i \in \mathbb {R}^{m}$ represent the input gene expression observed for object *i*, with *m* being the number of genes. In spatial transcriptomics data, each object *i* corresponds to a spatial location, often referred to as a spot. In contrast, for single-cell RNA-seq data, *i* indexes cells. To unify notation, we use $X_i$ to refer to the gene expression vector of an object regardless of whether it is a spot or a cell.

We define gene expression matrix $X_{n,m} \in \mathbb {R}^{n\times m}$, where *n* stands for the number of objects (spots or cells), and *m* for the number of genes. We consider two types of spatial prediction tasks based on different forms of spatial information as the response variable:

(i) Continuous coordinate prediction: Here, the goal is to predict the 2D spatial coordinates $Y_i\in \mathbb {R}^2$ of object *i*, using its gene expression vector $X_i$. This task is formulated as a multivariate regression problem by learning a function $f(X_i)$ that minimizes the mean squared error (MSE) between the predicted and observed spatial coordinates.
(ii) Domain recovery (classification): In this task, the objective is to predict the tissue domain (or layer) to which each object belongs. The response $Y_i$ is treated as a categorical variable indicating the domain label. For example, in the human cortex, the layers exhibit a clear ordinal structure, where layer labels (e.g., Layer 1-Layer 6 and White Matter) follow a biologically meaningful top-to-bottom spatial order. To reflect this ordinal structure in the model, we adopt a rank-consistent logistic loss function [6]. In contrast, for applications such as cancer studies where tissues are typically partitioned into non-ordered categories (e.g., tumor vs. normal regions), there is no meaningful order or hierarchy among the labels. In such cases, we use a standard cross-entropy loss function to model the classification task.

### KAN model

In this subsection, we introduce the specification of the Winnow-KAN model for $f(X_i)$. To introduce this model, we first illustrate an existing approach, the multiple-layer perceptron (MLP), that is constructed to perform spatial location prediction tasks. Specifically, a simple *L*-layer MLP network without bias with structure [$p_1, p_2, \cdot \cdot \cdot , p_L$], with $p_l$ stands for the number of nodes in layer *l* can be built to predict $Y_i$ by $f(X_i)$, given by

1 $$\begin{aligned} f(X_i) = \sigma (W_{L} \cdot \sigma (W_{L-1} \cdots \sigma (W_{2} \cdot \sigma (W_{1} \cdot X_i) ) ) ), \end{aligned}$$

where $W_l$ denotes a $p_l \times p_{l-1}$ matrix of trainable weights of the *l*th hidden layers, and $\sigma (\cdot )$ represents the activation function of the arguments there in the function $f(X_i)$ provides a strong approximation of the relationship described in the previous section, supported by the universal approximation theorem [12], which establishes its ability to approximate a wide range of functions. KAN model is distinct from MLP in that each node performs addition operations instead of multiplication by $W_l$. Specifically, unlike MLP whose activation functions are performed on the linear transformation of the values passed from the previous layers, the activation function in KAN, denoted as $\phi (x)$, consists of the weighted sum of trainable B-splines. Namely,

2 $$\begin{aligned} \phi (x) = w_{b}\cdot b(x) + w_s \cdot \textrm{spline}(x), \end{aligned}$$

where *b*(*x*) is a basis function of a generic argument *x*, that is often set as Silu$$(x) = \frac{x}{1 + e^{-x}}$$, and $$w_b $$ and $$w_s $$ are trainable weights representing the magnitude of specific significance for gene variables, and *spline*(*x*) is a function to impose non-linearity for *x*. Namely,

3 $$\begin{aligned} \textrm{spline}(x) = \sum _{k=1}^{K} c_k B_k (x), \end{aligned}$$

where $c_k$ is a trainable weight parameter, $B_k$ is a B-spline basis function [7], and *K* is the prespecified number of the basis functions, determined by a total grid size $G$ and spline order $\kappa$, such that $K = G + \kappa - 1$. Here, *G* is dynamically determined using input data from the previous layer. The B-spline basis functions $B_{j,k}(x)$ are specified recursively based on a grid of knots $\textbf{t} = \{t_j\}_{j=-\kappa }^{G+\kappa -1}$. Specifically, for a knot sequence $t_j \le t_{j+1}$, the 0th-order B-spline basis function is defined as

4 $$\begin{aligned} B_{j,0}(x) = {\left\{ \begin{array}{ll} 1 & \text {if } t_j \le x < t_{j+1}, \\ 0 & \text {otherwise}, \end{array}\right. } \end{aligned}$$

for $j = -\kappa , \ldots , G+\kappa -1$. Higher-order B-spline basis functions of order $k$ are computed recursively via

5 $$\begin{aligned} B_{j,k}(x) = \frac{x - t_j}{t_{j+k} - t_j} B_{j,k-1}(x) + \frac{t_{j+k+1} - x}{t_{j+k+1} - t_{j+1}} B_{j+1,k-1}(x), \end{aligned}$$

for $j = -k, \ldots , G-1$, where $k = 1, \ldots , \kappa$, and $\kappa$ is the spline order. The index $k$ in $B_k(x)$ of (3) corresponds to the basis function $B_{j,k}(x)$, with $j = k-1$, and the summation runs over $k = 1, \ldots , K$. The grid is updated by combining an adaptive grid, derived from the quantiles of the sorted input data $x_{\text {sorted}}$, and a uniform grid, ensuring even coverage across the data range. The updated grid is computed as a weighted average, controlled by a parameter $\epsilon$, and extended with additional knots to support the spline order $\kappa$. After updating the grid, the coefficients $c_k$ are recalculated via least-squares fitting to maintain consistency with the previous spline output.

Although both $w_s$ and $c_k$ are trainable, they exhibit a scaling redundancy: scaling $w_s$ by a constant and inversely scaling $c_k$ leaves the function output unchanged. From a strict identifiability perspective, this means $w_s$ is not uniquely determined. However, we intentionally retain this overparameterization because it serves a practical purpose in computations for variable selection. Specifically, we use $w_s$ as a proxy for gene importance. While non-identifiable, $w_s$ tends to concentrate around important input features during optimization. Overparameterization enlarges the solution space, allowing gradient-based optimizers to explore flat directions and converge to local minima that preserve function output while exhibiting useful sparsity patterns in $w_s$. This implicit bias toward sparsity makes $w_s$ a meaningful guide for selecting informative genes. In Section (B) of the Supplementary Materials, we formally analyze the scaling redundancy and its impact on optimization. We show that in the unconstrained setting, optimization occurs along scale-invariant rays, implicitly favoring sparsity in $w_s$. In contrast, normalization constrains parameters to a product manifold, eliminating this flexibility and promoting more uniform weight distributions. Empirical results confirm that retaining this redundancy enhances variable selection and improves downstream performance.

Therefore, for a KAN consisting of the structure as [$p_1, p_2, \cdot \cdot \cdot , p_L$], with $p_l$ stands for the number of nodes in layer *l*, the values passing from $(l-1)$th layer to *l*th layer of nodes is conducted by adding trainable B-splines and then summing at each node via (2), given by

6 $$\begin{aligned} \Phi _{l}(x) = \begin{pmatrix} \phi _{l,1,1}(\cdot ) & \phi _{l,1,2}(\cdot ) & \cdots & \phi _{l,1,p_{l-1}}(\cdot ) \\ \phi _{l,2,1}(\cdot ) & \phi _{l,2,2}(\cdot ) & \cdots & \phi _{l,2,p_{l-1}}(\cdot ) \\ \vdots & \vdots & \ddots & \vdots \\ \phi _{l,p_{l},1}(\cdot ) & \phi _{l,p_{l},2}(\cdot ) & \cdots & \phi _{l,p_{l},p_{l-1}}(\cdot ) \end{pmatrix} x, \end{aligned}$$

where *x* is a $p_{l-1}$-dimensional vector represents the values of nodes in layer ($l-1$), $\phi _{l,j,k}$ represents the operator in the form of (2) passing the *j*th node of *x* into the *k*th element of layer *l*. Then, a *L* layer KAN model can be written as

7 $$\begin{aligned} \textrm{KAN}(x) = (\Phi _L \circ \Phi _{L-1} \circ \cdots \circ \Phi _1)x. \end{aligned}$$

The trained model has two advantages. First, as indicated by Kolmogorov-Arnold representation theorem [15], the network has a strong approximation ability even with a smaller number of features, allowing it to represent any continuous function, regardless of its complexity. Second, it can also be used to identify the top genes that are most influential to the prediction by identifying the highest values of $w_s$. The details are described in Section 2.5.

### Architecture extension of KAN by integration activation functions

While nonlinear functions in KAN effectively capture complex relationships, they introduce numerical instability and gradient issues (e.g., vanishing or exploding gradients) during feature extraction [10]. To address this, we apply a ReLU activation function to each layer’s output, enhancing training convergence and nonlinear fitting capability for improved gene feature representation. Specifically, the activation in equation (2) is revised as

8 $$\begin{aligned} \phi (x) = \text {ReLU}(w_b \cdot b(x) + w_s \cdot \text {spline}(x)), \end{aligned}$$

where $\text {ReLU}(x) = \max (0, x)$. This modification emphasizes significant gradients, attenuates irrelevant updates, and provides sharper nonlinear boundaries compared to the original smoother formulation, boosting model robustness. In subsequent sections, we denote the original version of efficient-KAN [4] as default-KAN, contrasting it with our proposed Winnow-KAN.

### Winnow-KAN selector layer

A more complex (i.e., wider and deeper) KAN model is not necessarily optimal in the performance of prediction. Indeed, the additive characteristic of B-splines allows for a flexible representation of local patterns. In a higher-dimensional latent space, this flexibility may lead to an overemphasis on local variations rather than capturing broader global patterns, resulting in the issue of overfitting.

As a trade-off between information loss and model variability, lower dimensionality in the hidden latent space is often desired to ensure better generalization and interpretability. Therefore, it is essential to discard genes with minimal predictive power, which can be reflected by $w_s$. To address this, we introduce the Winnow-KAN selector layer, or Winnow block, which optimizes the balance between generalization and accuracy and provides a list of the most critical genes in the prediction. The introduction of the selector layer leads the prediction process to be in a two-stage scheme. In Stage 1, the Winnow-KAN model is fitted, where the first layer of the fitted model, referred to as the Winnow-KAN selection layer, is taken to identify the most important genes. In Stage 2, the model is refitted using the reduced dataset with fewer genes, enabling the final prediction tasks to be performed.

Specifically, the strategy for selecting genes depends on the dimension of layer 1, $$p_1$$ and their $$w_s$$; for the case where $$p_1 = 1 $$, i.e., only one node in layer *l*, we rank the variables according to their $$w_s$$ values in decreasing order. Then, the top *g* influential variables are given by those with the highest $$w_s$$. However, for cases where $$p_1>1$$, since we have multiple dimensions, we select the top genes that yield the highest significance coefficients in each node. Specifically, we define a value *t*, which represents the number of top variables selected from each dimension based on their significance coefficients. And let $$\mathcal {T}_{k}$$ be the top *t* variables in each node of layer 1, and then taking $$\bigcup _{k=1}^{p_1} \mathcal {T}_{k}$$, which consists of all the important variables across the $$p_1$$ nodes. However, since the coefficients of each dimension are not comparable, it is not feasible to provide the ranking of variable significance coefficients under this scenario. With the chosen top *g* genes kept, we obtain the sub-matrix $$M \in \mathbb {R}^{n\times g}$$ from *X*, which is then further taken as the training data for the second stage of model fitting. In Appendix B of Supplementary Material, we describe the mathematical rational of this weight-based gene selection.

### Model evaluation metrics

The evaluation metrics depend on the type of tasks performed. For the assessment of location prediction accuracy (task (i) in Section 2.2), we use the mean and median distance errors, as well as Pearson’s correlation. The mean distance error represents the average distance between all predicted and actual coordinate pairs, while the median distance error reflects the midpoint of these distance errors. To compare the predicted pairwise distances between cell pairs with their true pairwise distances, we compute the Pearson correlation coefficient to evaluate how well the model recovers the relative spatial relationships between cells. To evaluate the performance of our model in layer prediction (task (ii) in Section 2.2), we utilize both top-1 and top-2 accuracy metrics. Top-1 accuracy measures the percentage of instances where the model correctly predicts the exact layer for a given spot. In contrast, top-2 accuracy accounts for the percentage of spots where the model predicts either the correct layer or an adjacent layer, thus providing a more lenient assessment of the model’s performance.

## Results

### Layer recovery

To demonstrate the effectiveness of Winnow-KAN in identifying predictive genes and accurately recovering cortical layers in the human dorsolateral prefrontal cortex, even with a small number of genes, we performed a benchmark analysis using the LIBD dataset. This dataset includes manually annotated information of cortical layers in the brain, such as Layer 1 (L1), Layer 2 (L2), Layer 3 (L3), Layer 4 (L4), Layer 5 (L5), Layer 6 (L6), and White Matter (WM), providing a basis for assessing the accuracy of spatial domain recovery. In this study, we utilized tissue sections with IDs 151673, 151674, and 151675 as our training data, and a separate section 151676 as our test data for evaluation. To ensure data quality, we perform data preprocessing. First, we filtered out genes that were expressed in fewer than three cells, as well as mitochondrial genes, to eliminate potential noise. Following this, we normalized the gene expression data to ensure consistency and comparability across samples. We then applied a $log(x+1)$ transformation to the unique molecular identifier (UMI) counts, which helps stabilize variance and makes the data more suitable for downstream analysis. After these preprocessing steps, we retained 10,904 cells and 1,425 genes.

**Fig. 2:**
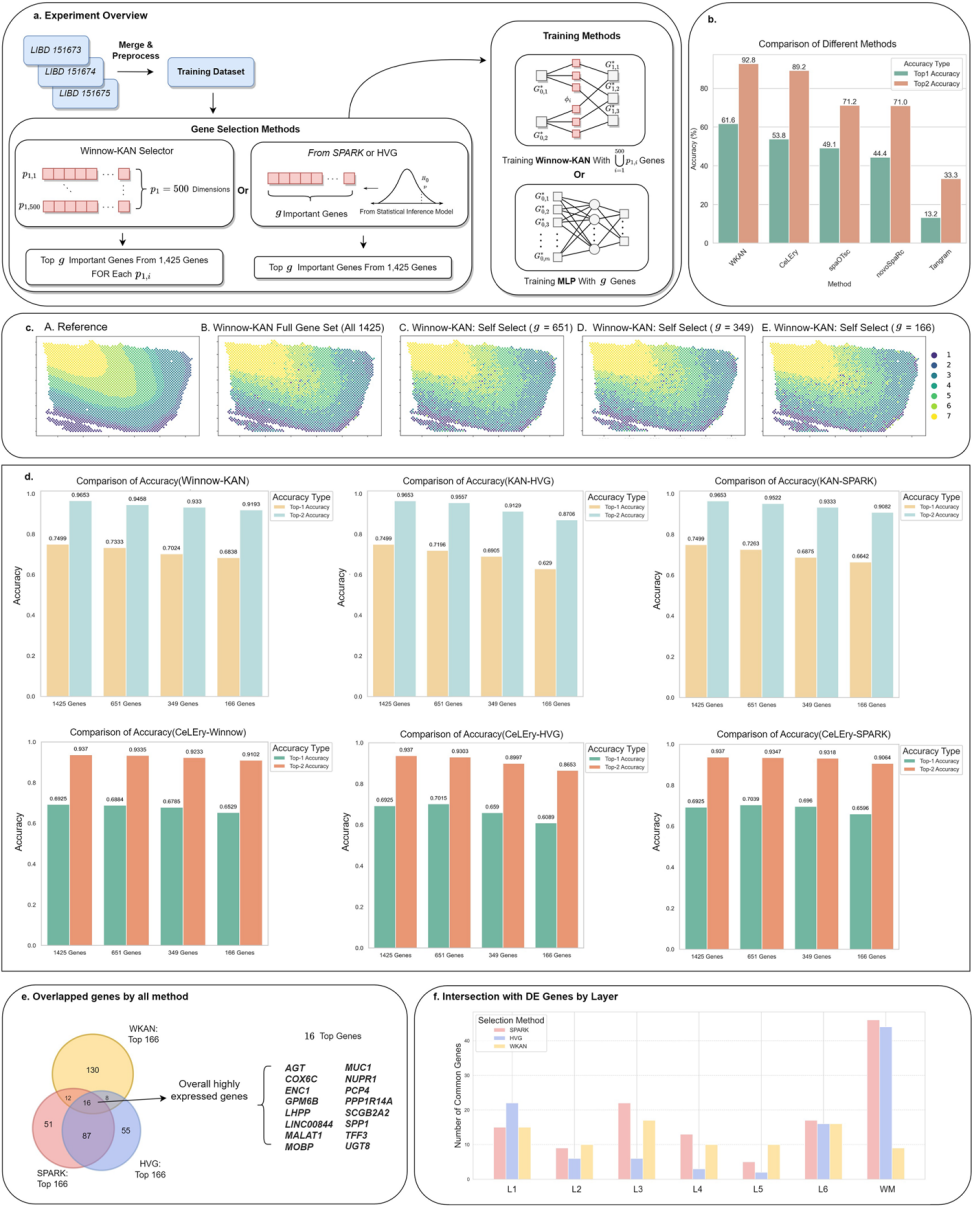
Layer recovery for cortical in the LIBD human data. **a** Three DLPFC samples (sample ID: 151673, 151674, and 151675) are merged and preprocessed to form the training dataset. The Winnow-KAN Selector is applied to each training spot $p_{1,i}$ (out of 500 total) to identify the top *g* most predictive genes from the full set of 1,425 genes. The union of selected genes across all training spots is used to train the Winnow-KAN model. In comparison, spatial variable genes and highly variable genes may be used as an alternative way of gene selection. The trained model is then tested on a held-out DLPFC sample (sample ID: 151676) for evaluation using either KAN or MLP. **b** Visualization of results for the top-1 and top-2 accuracies of five methods Winnow-KAN, CeLEry, spaOTsc, novoSpaRc, and Tangram trained on the sample 151673 and tested on the sample 151676 in LIBD dataset. **c** Layer-wise visualization of prediction on sample 151676 using the trained Winnow-KAN model with different numbers of selected genes. Panel A is the ground truth, and B-E are the predictions based on varying numbers of selected genes. **d** Barplot of top-1 and top-2 accuracies for HVG, Winnow-KAN, and SPARK with different numbers of selected genes trained with the Winnow-KAN model and CeLEry model. **e** Venn plot for the genes that each method selected. **f** Number of common genes between the three methods mentioned, with the layer-specific genes that are highly differentially expressed in L1-L6 and WM, respectively

We first examine the performance of Winnow-KAN in performing the layer recovery task. Here, we consider Winnow block’s dimension to be set as 500, and the hidden dimension of the full model structure is set to 500, 250, 100, 50, and 25 nodes for each layer, respectively (Fig. 2a). The input dimension corresponds to the number of genes in the expression matrix (1,425 genes), and the output dimension is 7, representing different layers (L1-L6 and WM). During the training phase, given the inherent order of the cortical layers, we incorporated this ordinal information by adopting COnsistent RAnk Logits (CORAL) loss [6]. We assign weights to the loss function of each spot based on the ratio of the counts of each layer to the total count, ensuring that the model appropriately balances the contributions from each layer. We compared Winnow-KAN’s power in prediction to using KAN with the genes selected from the Highly Variable Genes (HVG) obtained from the Seurat [26] method and spatial variable genes obtained from SPARK [28], respectively. Then, we further made the proposed KAN-based model in a closer comparison to CeLEry, which is an MLP-based predictive model. Similarly, we perform CeLEry on the genes selected by Winnow-block, HVG, and SPARK, respectively, yielding six methods in total in the comparisons (Fig. 2d and Fig S1 of the Supplementary Materials). Among those, Winnow-KAN outperforms the others, with the accuracy showing $74.99\%$, $73.33\%$, $70.24\%$, and $68.38\%$ for top-1 accuracy and $96.53\%$, $94.58\%$, $93.30\%$, and $91.93\%$ for top-2 accuracy, respectively, with 1425, 651, 349, and 166 genes retained. The accuracy of KAN with HVG is $74.99\%$, $71.96\%$, $69.05\%$, and $62.90\%$ for top-1 accuracy and $96.53\%$, $95.57\%$, $91.29\%$, and $87.06\%$ for top-2 accuracy. While the top-1 accuracy for KAN with SPARK is $74.99\%$, $72.63\%$, $68.75\%$, and $66.42\%$, top-2 accuracy are $96.53\%$, $95.22\%$, $93.33\%$, and $90.82\%$. The results demonstrate Winnow-KAN’s ability to effectively maintain the prediction performance, even with a reduced number of genes. Comparing the accuracies of the prediction made by Winnow-KAN with that of the MLP model, it is evident that Winnow-KAN outperforms the conventional MLP under the same set of selected genes. The results also show that our method consistently outperforms others in average accuracy, with the performance gap increasing as the number of genes decreases.

We further compare the influential genes chosen by Winnow-block with the spatial variable genes identified by SPARK and the high variable genes captured by Seurat (Fig. 2e). We observe that in the dataset of sample 151673, 16 genes exhibit high expression levels in all section methods. Winnow-KAN identified a substantial number of genes (130) that were not detected by other statistical methods, demonstrating its ability to provide a complementary perspective to traditional approaches in gene identification. To further explore the roles of the selected genes and assess their layer specificity, we analyzed the overlap between the selected genes and those showing the highest differential expression in each cortical layer (Fig. 2f). Specifically, for each brain layer, we identified a pool of 50 genes with the highest differential expression compared to all other layers, designating these as layer-specific genes. We then compared the overlap between the genes selected by Winnow-KAN, SPARK, and HVG with the layer-specific gene pools for each layer. Notably, the SPARK and HVG exhibit substantial overlap in gene selection within the white matter (WM), with fewer overlaps observed in layers such as L2 and L5. This is consistent with the significant differences in gene expression between the gray matter layers (L1-L6) and WM. In contrast, Winnow-KAN demonstrated a more uniform distribution of selected genes across layers, identifying fewer genes specific to WM. This suggests that Winnow-KAN requires fewer genes to effectively predict WM, demonstrating its efficiency in gene selection while achieving reasonable prediction performance. Notably, we compared our selected gene set with the top 50 DE genes for each layer, and our methods identified several critical genes that are not among those with the highest differential expression for each layer. For example, we identified *MAPK10* [8] and *MIF* [20], both are linked to AD pathology and central to neuroinflammation and cellular stress responses that are significantly dysregulated in AD. Another example is *SHANK3* [30], which is correlated with synaptic loss and cognitive decline. Furthermore, we identified *HSP90AA1* [2], a key chaperone protein that is implicated in the compromised protein misfolding and quality control mechanisms characteristic of AD.

We comment that the imbalanced sample size of training data for each category means there are chances that the important genes that are specific to certain small layers (such as L4) might be excluded by Winnow-selector, leading to the absence of L4. Therefore, we recommend adjusting the weight for the loss function according to the size of layers or other user-defined criteria. Another option is to fix the known set of genes that are specific to identify certain small categories to prevent them from being removed during the selection process.

To show the significance of the modification on the KAN architecture described in Section 2.5 by incorporating extra activation functions, we also conducted a comparative analysis to verify whether our modified KAN structure offers a genuine improvement in prediction accuracy over KAN in practical applications. Using a lightweight network architecture with dimensions [100, 50, 20], Winnow-KAN achieves superior performance. Specifically, Winnow-KAN attains a top-1 accuracy of $73.65\%$ and a top-2 accuracy of $95.13\%$, successfully predicting a significant portion of L4 outcomes. In contrast, the default KAN model achieved a top-1 accuracy of $69.89\%$ and a top-2 accuracy of $93.15\%$, while failing to predict L4 outcomes accurately. This illustrates the enhanced predictive performance of Winnow-KAN.

### 2D location recovery task

Building on the strong performance of Winnow-KAN in layer recovery demonstrated in Section 3.1, we extend our investigation to a more challenging task, identifying the precise 2D coordinates of each cell. To show that the Winnow-KAN can be implemented on spatial transcriptomics data generated from different platforms, we analyze a MERFISH Mouse Brain receptor map dataset [29]. We use the right brain of Slice 1 Replicate 2 for training, which contains 1,147 genes and 78,329 cells, and the whole brain of Slice 1 Replicate 1 to evaluate the performance of Winnow-KAN. After filtering out cells expressing fewer than 100 genes and 500 UMI counts, a total of 8,770 cells and 649 genes were retained for training. Following initial training for gene variable extraction, with a Winnow block with $p_1 = 1$ layer and subsequent five hidden layers, we set these chosen genes as the training dataset in a new model to test its validity. The overall Winnow block dimensions were configured as [649, 1, 150, 75, 25, 10, 2], with the input dimension corresponding to the number of gene variables, while the output dimension was set to 2, respectively corresponding to the coordinates of the x-axis and y-axis. For this test, we evaluated the quality of the selected genes using different subsets: 649 genes (the unaltered gene list), 80 genes, 40 genes, 20 genes, and 10 genes, respectively.

**Fig. 3:**
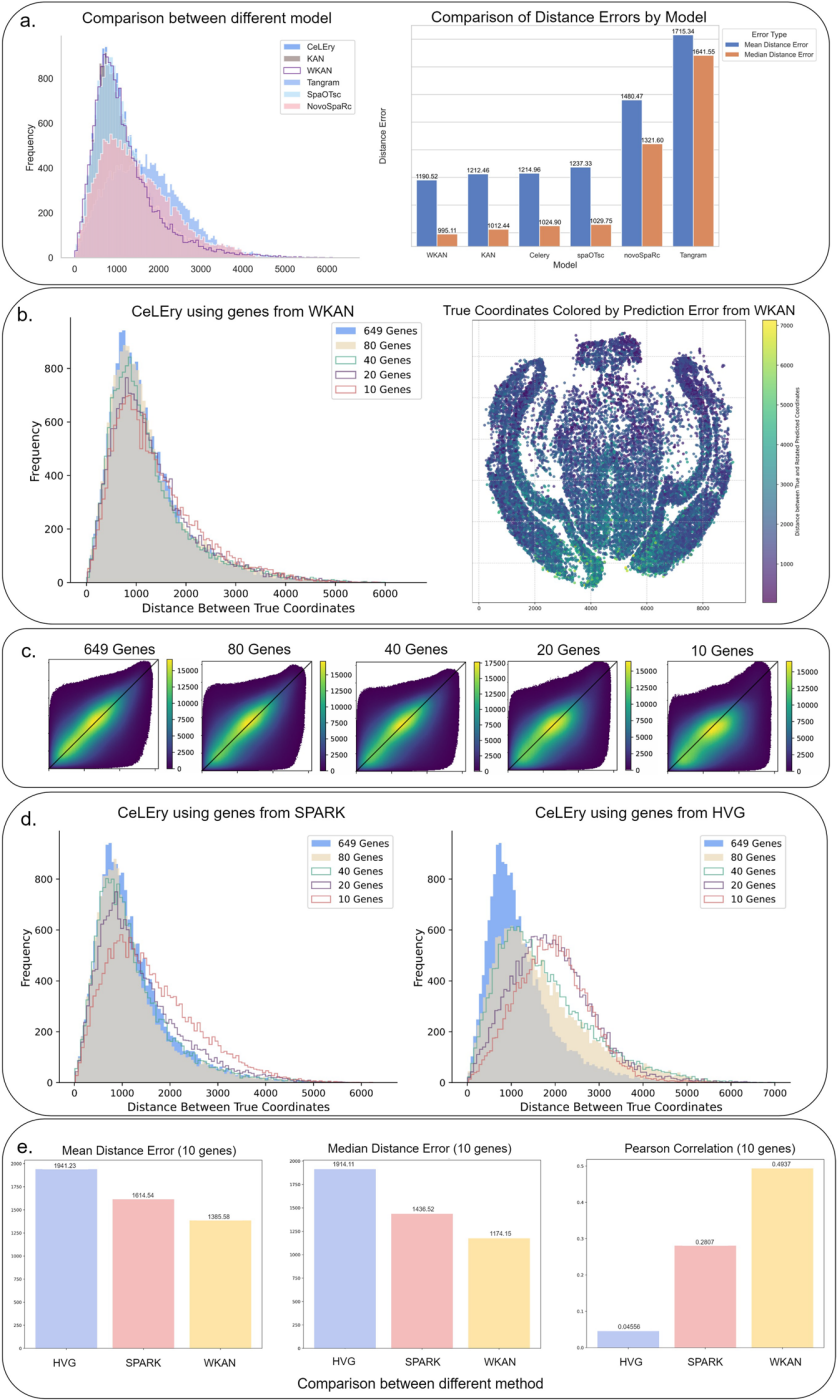
Coordinate prediction results on the MERFISH dataset. **a** Comparison of prediction errors across models. Left: Histogram of Euclidean distance errors (in pixels) between predicted and true coordinates, comparing Winnow-KAN, KAN, CeLEry, Tangram, spaOTsc, and NovoSpaRc. Right: Bar plot summarizing mean and median prediction errors for each model. **b** Evaluation of gene subsets selected by Winnow-KAN. Left: Distribution of prediction errors (in pixels) for the cells whose location is predicted using the model with different gene subsets (649, 80, 40, 20, 10 genes) selected by Winnow-KAN. Right: Spatial visualization of prediction errors across the tissue; color indicates the magnitude (in pixels) of prediction error per cell. **c** Scatter density plot illustrating the relationship between true and predicted pairwise distances for all cell pairs. The color gradient in the plot represents the density of cell pairs. **d** Comparison of prediction errors using genes selected by SPARK (left) and highly variable genes (HVG; right), evaluated using different subset gene sizes. **e** Summary of performance metrics for the models using 10-gene subsets as the predictors: mean distance error, median distance error, and Pearson correlation between predicted and true pairwise distances, comparing HVG, SPARK, and Winnow-KAN-selected genes

To test the significance of the chosen genes and demonstrate the robustness of our method across various implementations, we employed an MLP-based approach (i.e., in the same setting as CeLEry). Besides, we also compare with the default KAN, Tangram, spaOTsc, and novoSpaRc (Fig. 3a), we observe that Winnow-KAN outperforms both CeLEry and KAN by achieving a 2% increase in performance under the same model dimensions, and higher performance gain compared to Tangram, spaOTsc, and novoSpaRc. Of note, the improvement in comparing to the default KAN indicates that, consistent with the conclusions we demonstrated in Section 2.5, adding a ReLU loss function, similar to the role of residual connections in ResNet (He et al. [11]), after each layer of the KAN network can improve performance within relatively wide KAN model configurations. Furthermore, we achieved promising results with the preprocessed dataset of 649 genes, where our model reached a correlation of 0.6. This indicates that our improved method is effective in scenarios with the full number of input genes.

We further examine the prediction performance of Winnow-KAN with smaller sizes of genes sets (Fig. 3bc). When the number of genes was reduced to one-eighth of the original (80 genes), the model maintained fairly similar prediction accuracy. This indicates that nearly six-sevenths of the genes are redundant for representing the location information and can be safely excluded without significantly impacting the model’s performance. Furthermore, when the gene count was reduced from 80 to 40 and 20, the model still performed reasonably well, demonstrating its capability to handle tasks that do not require high precision. This highlights the robustness and efficiency of our method in minimizing redundant genes for spatial predictions.

To evaluate the accuracy of gene selection in our model, we compare it with SPARK and the HVG-based method from the Seurat package (Fig. 3d and Fig. S2bc in the Supplementary Materials). In these comparisons, our approach demonstrated superior performance compared to both alternatives. Notably, our method outperformed the others more significantly when the number of selected genes was smaller, highlighting its effectiveness in scenarios requiring a minimal gene set. In particular, when using only 10 genes as training data for the model (Fig. 3e), we observe that HVG performed the worst, with a Mean DE of 1941.23, a Median DE of 1914.11, and a Pearson’s correlation coefficient of 0.046. SPARK performed better but still fell short, with a Mean DE of 1614.54, a Median DE of 1436.52, and a relatively low Pearson’s correlation coefficient of 0.28. In contrast, our method achieved significantly better results, with reduced Mean DE (1385.58) and Median DE (1436.52), and a much higher Pearson’s correlation coefficient of 0.49. These findings highlight the superior accuracy and robustness of our approach to gene selection and predictive performance. In Fig. S2e of the Supplementary Materials, we present the spatial expression patterns of the top 10 genes identified by the Winnow Block as most influential for spatial prediction. These genes exhibit strong and distinct spatial localization across the tissue, demonstrating that Winnow-KAN effectively selects genes with meaningful spatial variation. This supports the model’s ability to uncover spatially informative genes that contribute to accurate cell location recovery and provide interpretable biological insights.

## Application

**Fig. 4:**
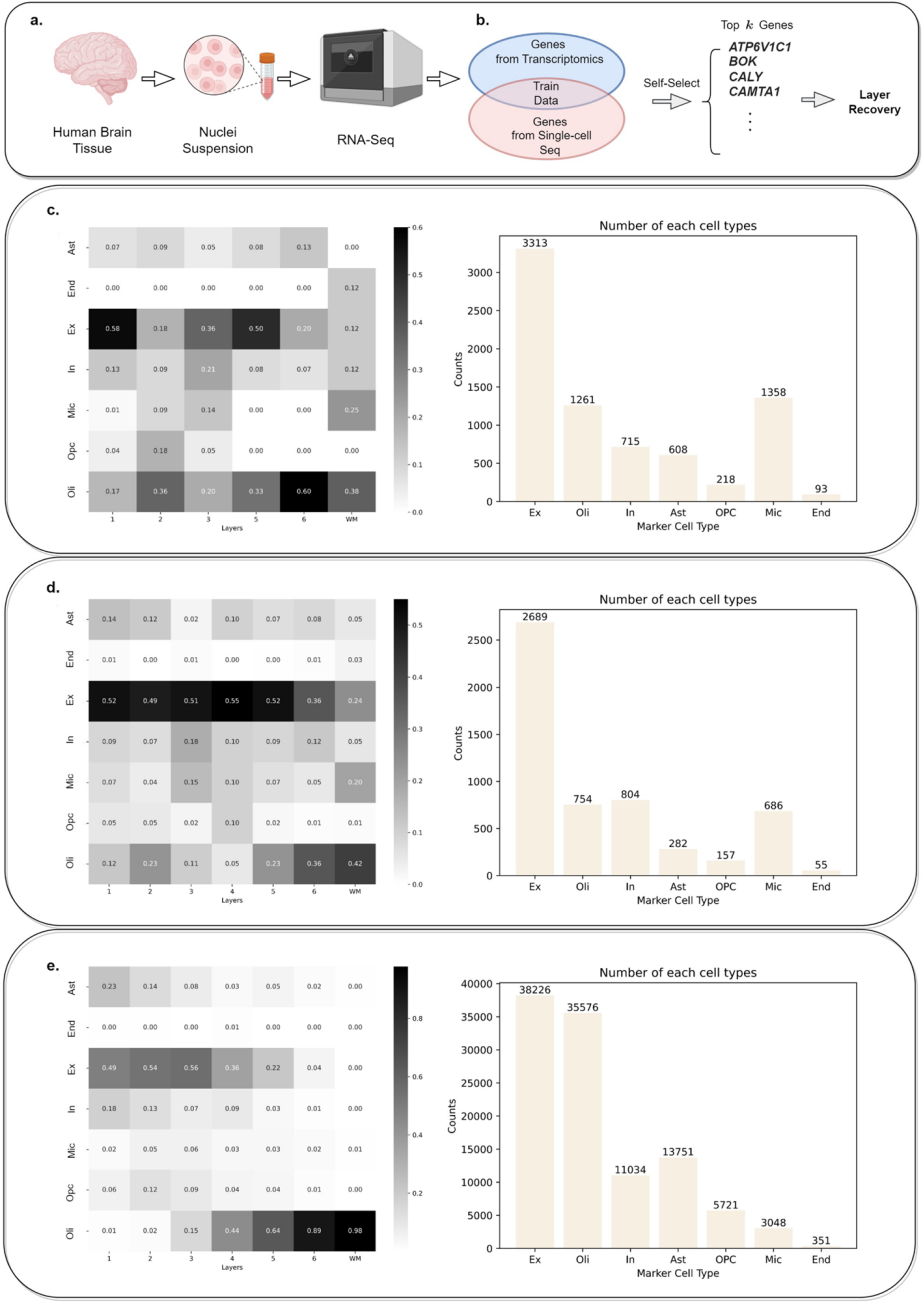
Application. **a** Workflow demonstrating the experimental process, starting from human brain tissue, nuclei suspension, RNA sequencing (RNA-seq), and subsequent gene selection. **b** Illustration of the integration of transcriptomic and single-cell sequencing data, highlighting self-selection of top marker genes for layer recovery. **c**–**e** Heatmaps representing the distribution of gene markers across different layers for distinct cell types (Ex, Oli, In, Ast, OPC, Mic, End). Adjacent bar plots depict the number of cells for each marker type

When recovering the spatial locations of single cells, a pre-trained model is highly desirable as it can be used directly without retraining. However, many existing methods, such as CeLEry, require the query data to be paired with reference data. This reliance on pairing limits the generalizability of these models, as those trained on one reference dataset often cannot be directly applied to a different query scRNA-seq dataset. This incompatibility arises from discrepancies in sequencing pipelines, which result in differences in the number of detected genes across single-cell RNA-seq datasets, making it challenging to use pre-trained models across datasets. To address this issue and improve the compatibility of pre-trained models, it is essential to use a smaller, standardized set of genes. In this section, we demonstrate how Winnow-KAN can be utilized to develop a pre-trained model by selecting a reduced number of key genes, which helps to enhance the model’s generalizability and compatibility with diverse single-cell datasets (Fig. 4a, b).

To illustrate the usage of Winnow-KAN in application to data collected from multiple sites, we use Winnow-KAN to recover the cell layer information for three datasets (Fig. 4c–e). The first two datasets (dataset ID: AD01302 and AD01304 in Lau et al. [18]) arise from a single-nucleus transcriptome analysis on Alzheimer’s disease. They are collected from female individuals with Braak stages 1–2 and 4–6, and ages 85.4 and 74.6, respectively. The third dataset comes from [23], which consists of snRNA-seq data generated for Alzheimer’s disease research. It includes 122,606 cells collected from the frozen middle frontal neocortex of 15 postmortem brains, designed to control for sex and age while capturing varying pathological conditions.

The predictions show consistent results across the three datasets despite being collected from different sources. Although the cell type compositions vary among the datasets, the predicted distributions of cell types within each layer are similar. For instance, astrocytes are present in L1, neurons are strongly represented in layers L1-L5, and oligodendrocytes are predominantly predicted in L6 and WM. These findings align with biological expectations and are consistent with those found by CeLEry, demonstrating the proposed method’s ability to recover layer information across multiple datasets simultaneously and that it can be generalizable to datasets with varying characteristics.

## Conclusion and discussion

In this study, we proposed the Winnow-KAN method, a novel approach designed to facilitate efficient prediction by outperforming traditional Multi-layer Perceptron (MLP) approaches even with a reduced set of genes. Through experimental benchmark studies on both feature selection and prediction as standalone components, we demonstrated that each component of Winnow-KAN contributes significantly to its superior performance. While prior works such as efficient-KAN and fast-KAN provide CUDA-enabled KAN implementations, Winnow-KAN is specifically adapted for spatial transcriptomics through its integration of a selector layer (Winnow Block) for gene interpretability and modifications to improve training stability on sparse, high-dimensional data.

While the genes selected by Winnow-KAN enhance predictive accuracy, they also provide supplementary insights that can complement existing findings. Interestingly, we observed that the genes identified by Winnow-KAN differ from those selected by traditional statistical models based on spatial variable gene (SVG) methods such as SPARK. While SVG typically emphasizes genes with distinct spatial patterns in their contrast of gene expression, Winnow-KAN prioritizes the predictability of the selected genes, and hence, these genes may not necessarily exhibit such precise patterns. This divergence stems from the limitations of the universal approximation theory in deep networks, as the final concatenated spline in Winnow-KAN lacks the granularity required for fine spatial details, making it less suitable for making interpretation on the spatial patterns. Nevertheless, the selected genes provide a unique alternative perspective for biological tasks that warrant further exploration, offering new opportunities to uncover insights that may be overlooked by traditional methods. It is also worth noting that some of these seemingly redundant genes may, in fact, be co-regulated or functionally related, possibly acting within the same biological pathway. While our current framework prioritizes parsimony and predictive power, future work could explore the network-level structure of these genes to better understand their coordinated roles in spatial organization.

In addition to the good prediction performance of Winnow-KAN, Winnow-KAN also demonstrates excellent transferability. To assess this transferability, we trained alternative MLP models within the CeLEry framework using the genes identified by Winnow-KAN. The genes selected by the selection layer of Winnow-KAN maintain reasonably robust performance when applied to alternative MLP-based models, such as CeLEry. This highlights the versatility and effectiveness of Winnow-KAN in optimizing gene selection for deep learning tasks in general.

Finally, Winnow-KAN reduces the computational complexity associated with handling high-dimensional gene expression data and achieves substantial efficiency gains without compromising accuracy. This makes it particularly well-suited for optimizing deep learning models in spatial transcriptomics, where computational resources are often a limiting factor.

A key limitation of coordinate-level prediction is that spatial coordinates are defined relative to each slide and are not inherently comparable across samples. Future work could incorporate tissue alignment methods, such as optimal-transport-based registration (Zeira et al. [32], Klein et al. [14]) or landmark-guided transformations [1], to enable cross-slide coordinate prediction in a geometrically meaningful way. More broadly, while Winnow-KAN is currently designed for anatomically matched tissues, extending its applicability to structurally heterogeneous samples, such as tumor sections from different regions or patients, remains an important direction. One promising strategy is to train on multiple aligned tissue sections while integrating additional contextual information, such as histological features or learned tissue embeddings, to account for structural variability. These extensions may ultimately enable Winnow-KAN to move beyond context-specific mapping and support more generalizable spatial inference.

## Supplementary Material

Supplementary file 1 (pdf 8985 KB)

## Data Availability

LIBD human DLPFC 10x Visium data: http://research.libd.org/spatialLIBD/. Mouse posterior brain 10x Visium data: https://support.10xgenomics.com/spatial-gene-expression/datasets/1.0.0/V1_Mouse_Brain_Sagittal_Posterior. Mouse Brain MERFISH data: https://doi.org/10.35077/act-bag. AD01302 &AD01304 Prefrontal cortex dataset: https://bmblx.bmi.osumc.edu/ssread/AD01302. These datasets are all publicly available; all datasets will be uploaded to GitHub.

## Code availability

The implementation of WinnowKAN can be accessed via: https://github.com/Yyuzrah/WinnowKAN.
