# Supplementary material for: Winnow-KAN: single-cell RNA-seq location recovery with small-gene-set spatial transcriptomics
Source: BMC Bioinformatics. 2025 Aug 12;26:209. doi: 10.1186/s12859-025-06243-9 (PMC12341082; doi:10.1186/s12859-025-06243-9)
Supplement: Supplementary file 1 — (pdf 8985 KB) [file 12859_2025_6243_MOESM1_ESM.pdf]

# A Supplementary Materials for "Winnow-KAN: Single-Cell RNA-seq Location Recovery with Small-Gene-Set Spatial Transcriptomics"

## A.1 Supplementary Figure 1

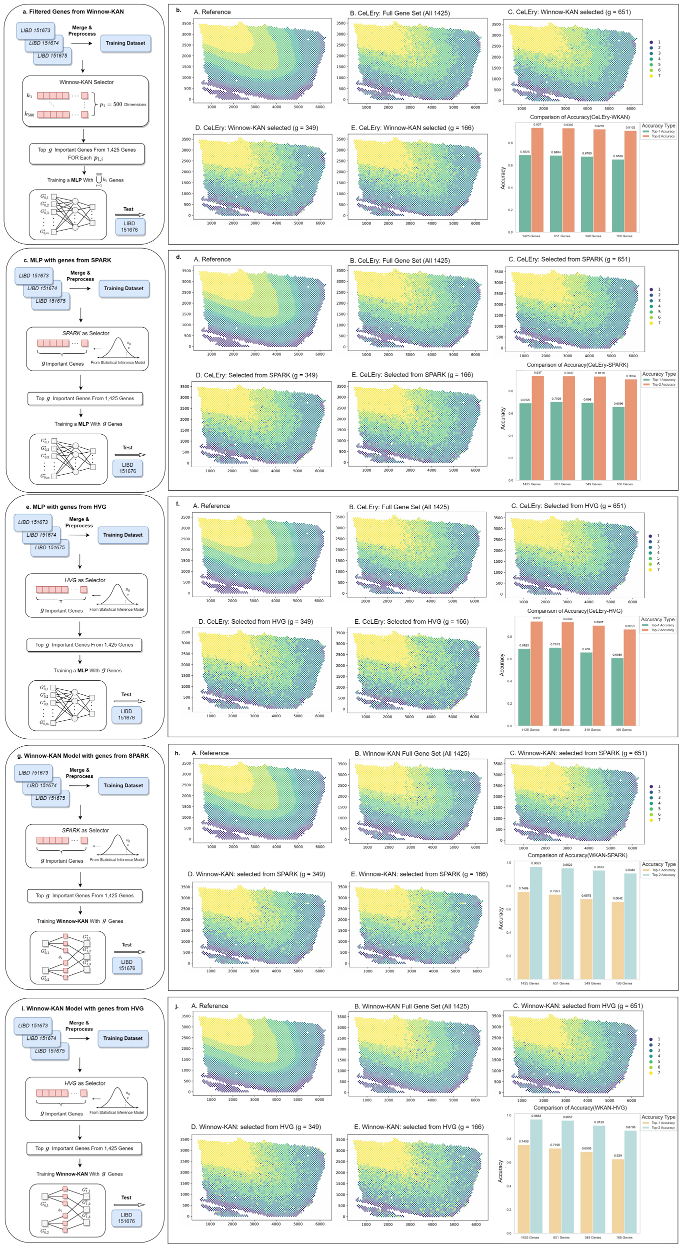

Figure S1: Evaluation of gene selection strategies for spatial layer recovery. (a,c,e,g,i) workflow of the experiment. (b,d,f,h,j) Genes were filtered using CeLEry and Winnow-KAN with various data subsets. Results display spatial reconstruction error across different layers using the filtered gene set and the impact of selected genes on recovery accuracy. Adjacent bar plots compare the performance of full and reduced gene sets.

## A.2 Supplementary Figure 2

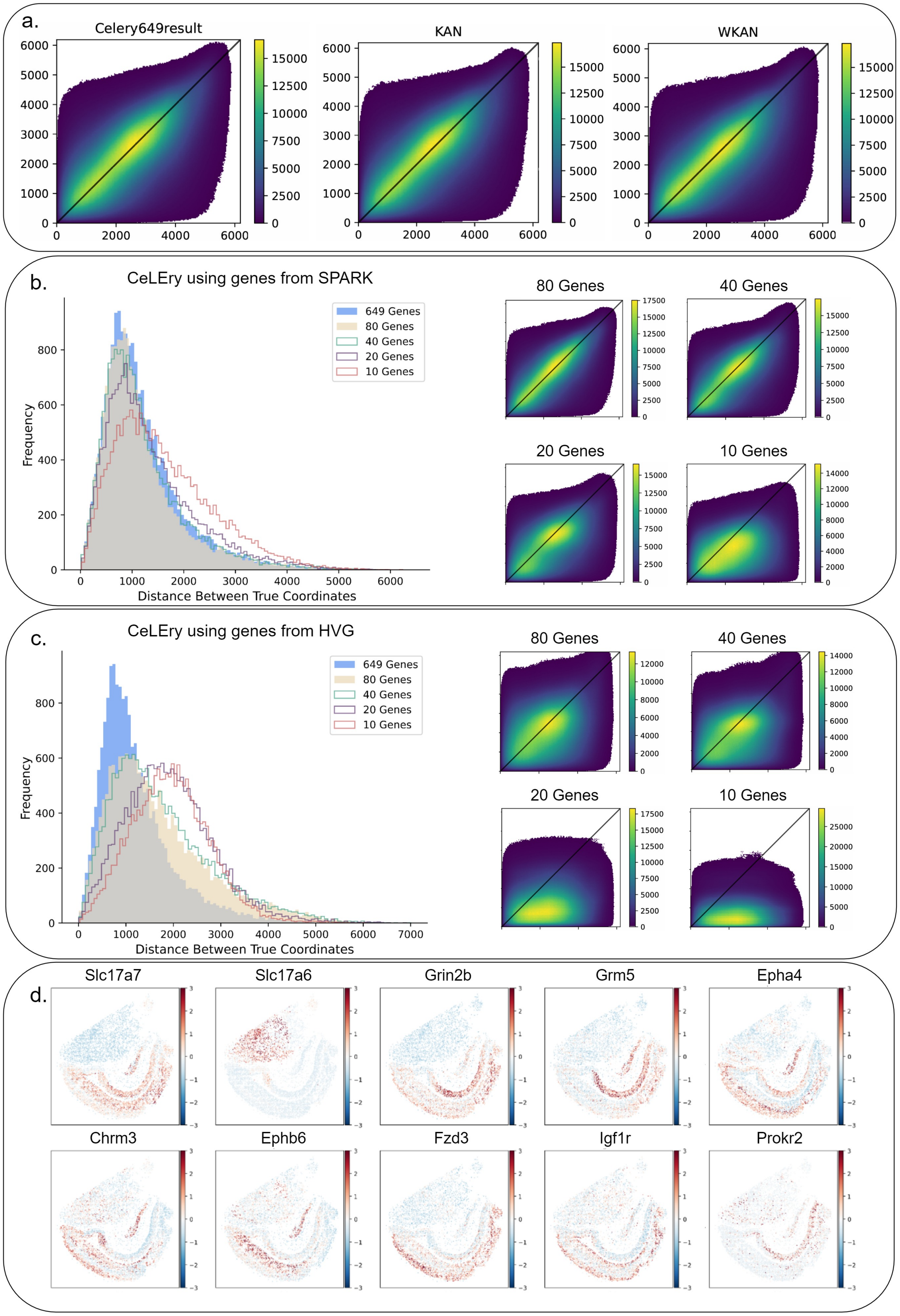

Figure S2: (a) Heatmaps comparing CeLEry's spatial coordinate predictions using 649 genes (baseline), KAN, and WKAN approaches, showing improved accuracy and reduced error variance across coordinates. (b) Performance evaluation of CeLEry using SPARK-filtered gene sets (649, 80, 40, 20, and 10 genes). The histogram shows error distributions between true and predicted coordinates, and heatmaps illustrate results for reduced subsets. (c) Similar evaluation using HVG-filtered gene sets, showing comparable trends in error reduction and reconstruction accuracy with decreasing gene subsets. (d) Visualization of the spatial gene expression of the 10 selected genes from Winnow-KAN, where spots colored in red are positively expressed, and blue spots are negatively expressed.

## A.3 Experiment on Comparing Training Time between KAN and Ours

We conducted experiments to compare the training efficiency of the original Kolmogorov-Arnold Network (KAN) and our method based on Efficient-KAN, across four toy regression tasks with increasing complexity. In Experiment 1, a dataset of 100 samples with one feature was generated by uniformly sampling $x$ from $[-1, 1]$ and computing $y = \sin(\pi x)$; both models used a two-layer architecture (1-5-1) with a grid size of 5 and spline order of 3, yielding approximately 50 and 45 parameters for the original and Efficient KAN, respectively, and training for 100 epochs with Adam (learning rate 0.001) and result back in 1.1424s for the original KAN and 0.4153s for the Efficient KAN. Next, Experiment 2 increased complexity with 1000 samples and two features sampled from $[-1, 1]^2$, targeting $y = x_1^2 + x_2^2$, using a two-layer structure (2-10-1) with the same grid and spline settings, approximately 200 and 180 parameters, respectively, where training took 2.7617s for the original KAN and 1.2215s for the Efficient KAN. Then, Experiment 3 scaled to 10,000 samples with five features from $[-1, 1]^5$ and $y = \sum_{i=1}^{5} x_i^2$, employing a three-layer network (5-10-10-1) with

roughly 500 and 450 parameters, respectively, completing in 53.9806s for the original KAN and 5.6666s for the Efficient KAN. Finally, Experiment 4 retained the same dataset but increased model complexity with a grid size of 10 and spline order of 5, raising parameter counts to approximately 1000 and 900, respectively, resulting in 160.3346s for the original KAN and 15.6581s for the Efficient KAN. Across all tasks, the Efficient KAN consistently reduced training times, leveraging optimized tensor operations despite identical theoretical complexity, with gains most pronounced in larger, more complex scenarios.

## A.4 Number of Overlapped Top Identified Genes in Tissue 151673 and 151674

Table 1: Number of Overlapped Genes in Different Top-$T$

| Top-T | 151673 | 151674 | # of Overlap | Percentage in 151673 (%) |
|---|---|---|---|---|
| 1 | 96 | 96 | 11 | 11.46 |
| 2 | 182 | 183 | 25 | 13.74 |
| 3 | 257 | 256 | 57 | 22.18 |
| 4 | 321 | 330 | 90 | 28.04 |
| 5 | 387 | 391 | 122 | 31.52 |
| 6 | 444 | 460 | 160 | 36.04 |
| 7 | 504 | 528 | 208 | 41.27 |
| 8 | 567 | 586 | 250 | 44.09 |
| 9 | 624 | 635 | 289 | 46.31 |
| 10 | 678 | 686 | 341 | 50.29 |

To evaluate the learning consistency of our method across distinct datasets, we trained identical neural network models, each with dimensions [100, 50, 25], on two separate datasets, 151673 and 151674. Post-training, variable selection was conducted by ranking 1425 genes per node based on the absolute values of their weights across all 100 nodes. For each node, we selected the top-$t$ genes, with $t$ ranging from 1 to 10, and computed the intersection of these selections for each model. The consistency was then assessed by determining the intersection of gene sets selected from the two models. As illustrated in Table 1, the intersection size increases with $t$, from 11 genes at $t = 1$ (with union sizes of 96 for both models) to 341 genes at $t = 10$ (with union sizes of 678 and 686 for datasets 151673 and 151674, respectively).

# B Justification and Proof for Scaling Redundancy

As aforementioned, the $W_s$ and $C_k$ scale redundancy can be a concern that is harmful to the identifiability of our model's interpretable ability. Here we first set this problem: We define the solution parameter space for the first layer (layer 1) as $\Theta = \mathbb{R}^{p_1 \times m} \times \mathbb{R}^{p_1 \times m \times K} \times \mathbb{R}^{p_1 \times m}$, where $m$ is the number of input genes (variables) to layer 1, $K$ is the number of B-spline basis functions for each spline($\cdot$) term, and $p_1$ is the number of nodes in layer 1. The parameter space excludes $w_b$ for simplicity of analysis, focusing on the $W_s$ and $C_k$ redundancy.

The functional component $\phi_{l,j,k}(\cdot)$ in equation (4) corresponds to $\phi(x)$ in equation (2), specifically for layer $l = 1$. Thus, for the connection from the $i$-th input gene to the $m$-th node of layer 1, the relevant term from equation (2) and (3) is:

$$W_{s,mi} \cdot \text{spline}_{mi}(x_i) = W_{s,mi} \sum_{k=1}^{K} C_{mik} B_k(x_i)$$

Here, $W_{s,mi}$ is the trainable weight $w_s$ for the spline part of the activation function connecting the $i$-th input gene to the $m$-th node in layer 1, and $C_{mik}$ is the $k$-th trainable weight $c_k$ for the B-spline basis function $B_k(x_i)$ in that specific activation function.

For any $\alpha_{mi} \in \mathbb{R}_{>0}$, the following transformations keep $\phi_{1,m,i}(\cdot)$ (which corresponds to

$W_{s,mi} \sum_{k=1}^{K} C_{mik} B_k(x_i)$ plus the $w_b$ term) invariant with respect to the spline part:

$$\begin{cases} W_{s,mi} & \mapsto \alpha_{mi} W_{s,mi} \\ C_{mik} & \mapsto \frac{1}{\alpha_{mi}} C_{mik} \quad \forall k. \end{cases}$$

In the experiment examining scaling redundancy, we applied the normalization for $\{C_{mik}\}_{k=1}^{K}$. The normalization constraint is defined as:

$$\text{norms}_{mi} = \left\| \{C_{mik}\}_{k=1}^{K} \right\|_2 = \sqrt{\sum_{k=1}^{K} C_{mik}^2}$$

Then:

$$W_{s,mi} \leftarrow \text{norms}_{mi}$$

and then:

$$C'_{mik} \leftarrow \frac{C_{mik}}{\text{norms}_{mi} + \epsilon}$$

where $\epsilon$ is a small constant for numerical stability. In this way, $W_{s,mi}$ is effectively set as the $L_2$-norm of $\mathbf{C}_{mi} = \{C_{mik}\}_{k=1}^{K}$, and $C'_{mik}$ are normalized such that $\|\{C'_{mik}\}_{k=1}^{K}\|_2 = 1$. Under this setting, the parameters we actually train for the spline part are $W_{s,mi}$ (as the norm of $\mathbf{C}_{mi}$) and $C'_{mik}$ (as the direction vector of $\mathbf{C}_{mi}$).

Let $f_{mi}(\mathbf{x}_i; w, \mathbf{c})$ be a functional mapping for the spline component connecting input gene $i$ to output node $m$. Here $w := W_{s,mi} \in \mathbb{R}$ and $\mathbf{c} := (C_{mi1}, \ldots, C_{miK}) \in \mathbb{R}^K$ are parameters for a single component. Assume $f_{mi}$ possesses a scaling invariance:

$$f_{mi}(\mathbf{x}_i; w, \mathbf{c}) = f_{mi}(\mathbf{x}_i; \alpha w, \alpha^{-1}\mathbf{c}) \quad \forall \alpha \in \mathbb{R}_{>0}$$

Let $L(\Theta)$ be the loss function to be minimized, where $\Theta = ((W_{s,11}, \mathbf{C}_{11}), \ldots, (W_{s,p_1 m}, \mathbf{C}_{p_1 m}))$ is the collection of all parameters for the spline components in the first layer. The total number of such components is $N = p_1 \times m$. We analyze the distribution of magnitudes $\{|W_{s,mi}|\}_{(m,i)\in\{1,\ldots,p_1\}\times\{1,\ldots,m\}}$ at optimal $\Theta^*$. For simplicity, we denote these magnitudes as $\{|w_j|\}_{j=1}^{N}$ where $j$ is a flattened index for $(m, i)$.

## B.1 Measures of Distribution Uniformity

**Definition B.1** (Uniformity Measure)**.** For a vector of non-negative magnitudes $\mathbf{a} = (|w_1|, \dots, |w_N|) \in \mathbb{R}^N_{\geq 0}$, we define a uniformity measure $U(\mathbf{a})$ as the ratio of $L_1$ to $L_2$ norms:

$$U(\mathbf{a}) \coloneqq \frac{\|\mathbf{a}\|_1}{\|\mathbf{a}\|_2} = \frac{\sum_{j=1}^N |w_j|}{\sqrt{\sum_{j=1}^N w_j^2}}$$

For a given $L_2$ norm $\|\mathbf{a}\|_2 = C > 0$, $U(\mathbf{a})$ is maximized when all $|w_j|$ are equal ($|w_j| = C/\sqrt{N}$ for all $j$), yielding $U(\mathbf{a}) = \sqrt{N}$. $U(\mathbf{a})$ is minimized when only one $|w_j|$ is non-zero ($|w_j| = C$ for some $j$), yielding $U(\mathbf{a}) = 1$. Thus, a larger $U(\mathbf{a})$ indicates a more uniform (less sparse) distribution of magnitudes.

## B.2 Analysis of Parameter Spaces and Optimization Dynamics

**Lemma B.1** (Implicit Bias towards Sparse $W_s$ in Unconstrained Euclidean Space)**.** *Consider optimization of $L(\Theta)$ in the unconstrained Euclidean parameter space $\mathcal{P}_{unc} = (\mathbb{R} \times \mathbb{R}^K)^N$. Let $\Theta^*_{unc}$ be an optimal solution. The scaling invariance of $f_{mi}$ implies that for any optimal functional contribution $f^*_{mi}$, there exists an infinite set of parameter pairs $(W_{s,mi}, \mathbf{C}_{mi})$ satisfying this contribution. An optimizer exploiting this freedom can find solutions where, for components $j$ making significant contributions, $|W_{s,j}|$ are driven to very large values, leading to a small $U(|W_{s,1}|, \dots, |W_{s,N}|)$.*

*Proof.* Let $h_j(\mathbf{x}_j) \coloneqq W_{s,j}\mathbf{C}_j^\top B(\mathbf{x}_j)$. The invariance means $h_j(\mathbf{x}_j)$ is preserved for $(W_{s,j}, \mathbf{C}_j) \mapsto (\alpha W_{s,j}, \alpha^{-1}\mathbf{C}_j)$. The loss $L$ is constant along these scaling rays for each component $j$. This implies that the gradient $\nabla L$ has a zero component along these directions for each component's parameter pair $(W_{s,j}, \mathbf{C}_j)$. In overparameterized neural networks, gradient descent (and its variants) often exhibits an implicit bias. When multiple equivalent solutions exist (i.e., loss is flat along certain directions), the optimizer implicitly selects among them based on its dynamics. In this Euclidean setting, the optimizer can increase $|W_{s,j}|$ arbitrarily by choosing an inversely proportional $\|\mathbf{C}_j\|_2$, without changing the loss. This freedom allows the optimizer to concentrate the "effective magnitude" for crucial components into extreme $|W_{s,j}|$ values. This phenomenon is a known implicit regularization property of gradient

descent in overparameterized models, favoring solutions where a few parameters are significantly larger than others to achieve the desired functional output. This leads to a highly concentrated distribution of $\{|W_{s,j}|\}$, thus minimizing $U(\cdot)$. □

**Lemma B.2** (Geometric Constraint and Loss of Scaling Freedom)**.** *The constraint $W_{s,j} = \|\mathbf{C}_j\|_2$ and the subsequent normalization $\mathbf{C}_j \mapsto \mathbf{C}_j/\|\mathbf{C}_j\|_2$ (for $\mathbf{C}_j \neq \mathbf{0}$) maps the parameter space for each component $j$ to a manifold $\mathcal{M}_j = \mathbb{R}_{\geq 0} \times \mathbb{S}^{K-1}$, where $\mathbb{S}^{K-1}$ is the $(K-1)$-dimensional unit sphere. The optimization is then performed on the product manifold $\mathcal{P}_{con} = \prod_{j=1}^{N} \mathcal{M}_j$. This constraint explicitly eliminates the independent scaling freedom of $W_{s,j}$ and $\mathbf{C}_j$.*

*Proof.* For any component $j$, the original parameters are $(W_{s,j}, \mathbf{C}_j) \in \mathbb{R} \times \mathbb{R}^K$. We analyze the case where $\mathbf{C}_j \neq \mathbf{0}$, as $\mathbf{C}_j = \mathbf{0}$ corresponds to a trivial component. The constraint imposes the structure $W_{s,j} = \|\mathbf{C}_j\|_2$. By parameterizing the vector $\mathbf{C}_j$ by its magnitude and direction, we can define a new set of parameters $(W'_{s,j}, \mathbf{C}'_j)$ where $W'_{s,j} = \|\mathbf{C}_j\|_2$ and $\mathbf{C}'_j = \mathbf{C}_j/\|\mathbf{C}_j\|_2$. The parameter space for $(W'_{s,j}, \mathbf{C}'_j)$ is thereby restricted to $\mathbb{R}_{\geq 0} \times \mathbb{S}^{K-1}$, which we denote as the manifold $\mathcal{M}_j$.

Recall that the scaling equivalence class for a non-trivial component is given by $\{(\alpha W^0_{s,j}, \alpha^{-1}\mathbf{C}^0_j) \mid \alpha \in \mathbb{R}_{>0}\}$ for some initial parameters $(W^0_{s,j}, \mathbf{C}^0_j)$. The constraint forces us to choose a unique representative from this class. Specifically, for any member $(W_{s,j}, \mathbf{C}_j)$ of the class, where $W_{s,j} = \alpha W^0_{s,j}$ and $\mathbf{C}_j = \alpha^{-1}\mathbf{C}^0_j$, the reparameterization yields:

$$W'_{s,j} = \|\mathbf{C}_j\|_2 = \|\alpha^{-1}\mathbf{C}^0_j\|_2 = \alpha^{-1}\|\mathbf{C}^0_j\|_2$$

$$\mathbf{C}'_j = \frac{\mathbf{C}_j}{\|\mathbf{C}_j\|_2} = \frac{\alpha^{-1}\mathbf{C}^0_j}{\alpha^{-1}\|\mathbf{C}^0_j\|_2} = \frac{\mathbf{C}^0_j}{\|\mathbf{C}^0_j\|_2}$$

The directional component $\mathbf{C}'_j$ is invariant for all members of the equivalence class. The magnitude component $W'_{s,j}$ now explicitly depends on the scaling factor $\alpha$. Thus, the one-dimensional degree of freedom associated with $\alpha$ is not eliminated but is absorbed into the magnitude parameter $W'_{s,j}$. The independent scaling freedom between $W_{s,j}$ and $\mathbf{C}_j$ is removed because $W_{s,j}$ is now strictly determined by $\|\mathbf{C}_j\|_2$.

Geometrically, this constraint collapses the entire ray representing an equivalence class in the original parameter space onto a single point in the new constrained space $\mathcal{M}_j$. In the

unconstrained setting, the loss function is constant along these rays, creating "flat directions" in the loss landscape. By enforcing $W_{s,j} = \|\mathbf{C}_j\|_2$, any infinitesimal change $\delta\mathbf{C}_j$ induces a deterministic change in $W_{s,j}$:

$$\delta W_{s,j} = \delta(\|\mathbf{C}_j\|_2) = \frac{\mathbf{C}_j \cdot \delta\mathbf{C}_j}{\|\mathbf{C}_j\|_2} = \mathbf{C}'_j \cdot \delta\mathbf{C}_j$$

This coupling between the magnitude $W_{s,j}$ and the vector $\mathbf{C}_j$ removes the flat directions. Movement in the parameter space is no longer "free" along the scaling dimension; any change in scale now corresponds to a specific movement on the manifold $\mathcal{M}_j$ with a potential change in the loss. The optimization over the entire model is therefore performed on the product manifold $\mathcal{P}_{\text{con}} = \prod_{j=1}^{N} \mathcal{M}_j$, where each component's parameterization is unique and devoid of the original scaling redundancy. □

**Theorem B.1** (Dispersion of Optimal Magnitudes under Spherical Constraint)**.** *Let* $\mathbf{w}^*_{unc} = (|W^*_{s,1}|, \ldots, |W^*_{s,N}|)$ *be the vector of optimal magnitudes from the unconstrained optimization, and* $\mathbf{w}^*_{con} = (|W'^*_{s,1}|, \ldots, |W'^*_{s,N}|)$ *from the constrained optimization. Then, the uniformity measure satisfies* $U(\mathbf{w}^*_{con}) \geq U(\mathbf{w}^*_{unc})$*, implying a more dispersed distribution of magnitudes in the constrained case.*

*Proof.* In the unconstrained problem $\mathcal{P}_{\text{unc}}$, Lemma 1 implies that for any optimal solution, an equivalent solution can be constructed by arbitrarily scaling the magnitudes $|W_{s,j}|$. An optimizer can exploit this by driving a subset of magnitudes to be exceedingly large while others remain small. This leads to a highly concentrated magnitude distribution, thus yielding a minimal uniformity measure $U(\mathbf{w}^*_{\text{unc}})$.

In the constrained problem $\mathcal{P}_{\text{con}}$, Lemma 2 removes this scaling freedom by enforcing the spherical constraint, which ties the magnitude directly to the Euclidean norm, $|W'^*_{s,j}| = \|\mathbf{C}^*_j\|_2$. The optimization now occurs on the product manifold $\mathcal{P}_{\text{con}}$, which is compact. The geometry of the manifold inherently regularizes the solution. Increasing the norm $\|\mathbf{C}^*_j\|_2$ for one component while minimizing a global loss function is geometrically penalized by the manifold's structure, as updates are projected onto the tangent space at each point. This discourages the concentration of magnitudes seen in the unconstrained case.

Consequently, to minimize the overall loss, the optimization process on $\mathcal{P}_{\text{con}}$ favors a more equitable distribution of norms $\|\mathbf{C}^*_j\|_2$ across all components. A more uniform distribution

of magnitudes $\{|W'^*_{s,j}|\}$ necessarily results in a greater or equal uniformity value. Therefore, $U(\mathbf{w}^*_{\text{con}}) \geq U(\mathbf{w}^*_{\text{unc}})$. $\square$

## B.3 Experiment part

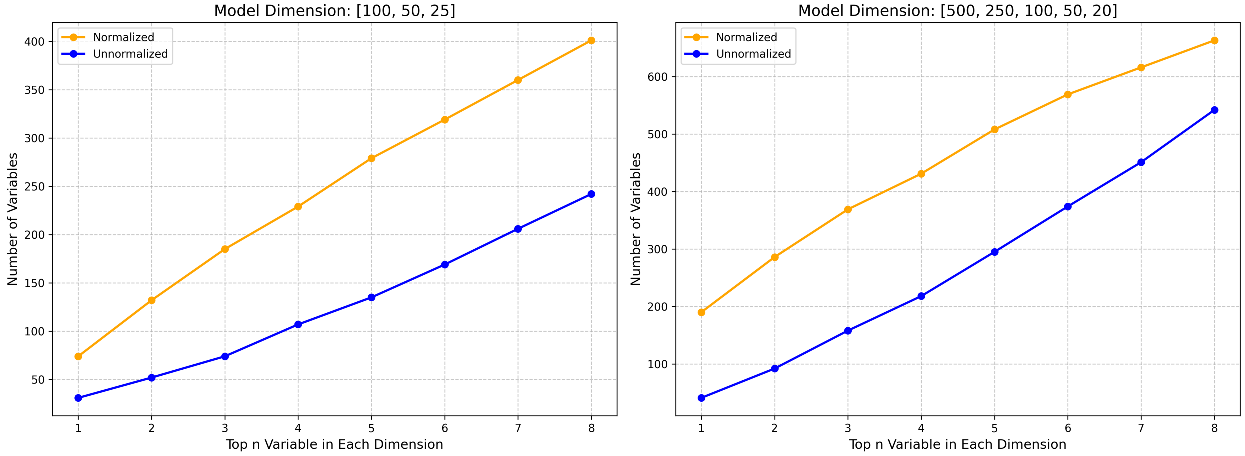


Figure S3: In this figure, we present the results from two distinct KAN models, configured with different layer parameters, to substantiate our aforementioned proof. Specifically, the left panel depicts a model with layer dimensions [100, 50, 25], while the right panel illustrates a model with layer dimensions [500, 250, 100, 50, 25]. Our analysis reveals that in the unnormalized scenario, the number of unions is notably smaller, which indicates a more concentrated distribution of top variables. In contrast, the normalized scenario exhibits a larger number of unions, suggesting a less concentrated distribution of top variables.

Given that our variable selection method fundamentally aims to identify more representative dimensions (i.e., variables) within the solution space, the introduction of normalization, which renders the distribution more uniform, may conversely impair our results. Below, we present the genes selected by the aforementioned two distinct models, along with their corresponding experimental outcomes.

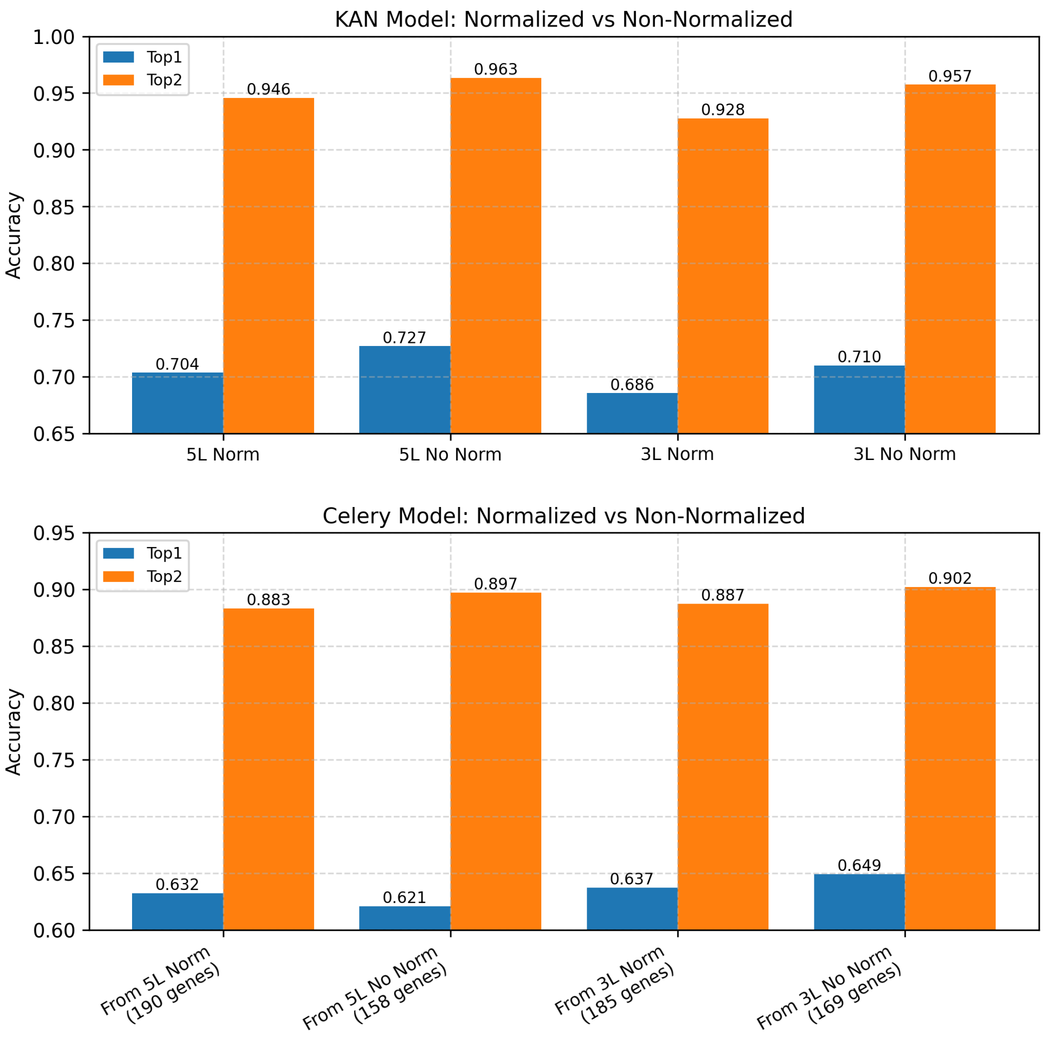


Figure S4: This figure illustrates the impact of normalization on the accuracy of KAN and Celery models. For both the 5-layer and 3-layer KAN models, the non-normalized approach consistently achieves higher Top-1 and Top-2 accuracies, with improvements ranging from 0.0175 to 0.0297. In Celery models using genes from the 3-layer KAN, non-normalization enhances performance, with gains of 0.0120 in Top-1 and 0.0146 in Top-2. However, for Celery models using genes from the 5-layer KAN, normalization increases Top-1 accuracy by 0.0114, while non-normalization boosts Top-2 accuracy by 0.0137.

The results indicate that incorporating normalization does not enhance performance. Al-

though it theoretically resolves the issue of scale redundancy, it actually diminishes the effectiveness of variable selection and negatively affects the performance of the KAN model.
